# The complex relationship between iron status and anemia in pregnant and postpartum women in India: Analysis of two Indian study cohorts of uncomplicated pregnancies

**DOI:** 10.1002/ajh.27059

**Published:** 2023-08-31

**Authors:** Manisha Nair, Saswati S. Choudhury, Anjali Rani, Carolin Solomi, Swapna D. Kakoty, Robin Medhi, Sereesha Rao, Pranabika Mahanta, Farzana Zahir, Indrani Roy, Shakuntala Chhabra, Gitanjali Deka, Bina Minz, Rupanjali Deka, Charles Opondo, David Churchill, Samira Lakhal‐Littleton, Elizabeta Nemeth, the MaatHRI collaboration

**Affiliations:** ^1^ National Perinatal Epidemiology Unit, Nuffield Department of Population Health University of Oxford Oxford UK; ^2^ Department of Obstetrics and Gynaecology Gauhati Medical College and Hospital Guwahati India; ^3^ Institute of Medical Sciences Banaras Hindu University, Banaras Hindu University Campus Varanasi India; ^4^ Makunda Christian Leprosy and General Hospital Karimganj India; ^5^ Department of Obstetrics and Gynaecology Fakhruddin Ali Ahmed Medical College and Hospital Barpeta India; ^6^ Department of Obstetrics and Gynaecology Silchar Medical College and Hospital Silchar India; ^7^ Department of Obstetrics and Gynaecology Jorhat Medical College and Hospital Jorhat India; ^8^ Department of Obstetrics and Gynaecology Assam Medical College Dibrugarh India; ^9^ Nazareth Hospital Shillong India; ^10^ Department of Obstetrics and Gynaecology Mahatma Gandhi Institute of Medical Sciences Sevagram India; ^11^ Department of Obstetrics and Gynaecology Tezpur Medical College Tezpur India; ^12^ Sewa Bhawan Hospital Society Basna India; ^13^ MaatHRI Srimanta Sankaradeva University of Health Sciences Guwahati India; ^14^ Department of Medical Statistics London School of Hygiene and Tropical Medicine London UK; ^15^ The Royal Wolverhampton Hospitals NHS Trust University of Wolverhampton Wolverhampton UK; ^16^ Department of Physiology, Anatomy & Genetics University of Oxford Oxford UK; ^17^ David Geffen School of Medicine & the UCLA Center for Iron Disorders University of California Los Angeles California USA

## Abstract

Low hemoglobin is widely used as an indicator of iron deficiency anemia in India and other low‐and‐middle income counties, but anemia need not accurately reflect iron deficiency. We examined the relationship between hemoglobin and biomarkers of iron status in antenatal and postnatal period. Secondary analysis of uncomplicated singleton pregnancies in two Indian study cohorts: 1132 antenatal women in third trimester and 837 postnatal women 12–72 h after childbirth. Associations of hemoglobin with ferritin in both data sets, and with sTfR, TSAT, and hepcidin in the postnatal cohort were examined using multivariable linear regression. Multinomial logistic regression was used to examine the association between severity of anemia and iron status. Regression models were adjusted for potential confounders. Over 55% of the women were anemic; 34% of antenatal and 40% of postnatal women had low ferritin, but 4% antenatal and 6% postnatal women had high ferritin. No evidence of association between hemoglobin and ferritin was observed (antenatal: adjusted coefficient [aCoef] −0.0004, 95% confidence interval [CI] −0.001, 0.001; postnatal: aCoef −0.0001, 95% CI −0.001, 0.001). We found a significant linear association of hemoglobin with sTfR (aCoef −0.04, 95% CI −0.07, −0.01), TSAT (aCoef −0.005, 95% CI −0.008, −0.002), and hepcidin (aCoef 0.02, 95% CI 0.02, 0.03) in postnatal women. Likelihood of low ferritin was more common in anemic than non‐anemic women, but high ferritin was also more common in women with severe anemia in both cohorts. Causes of anemia in pregnant and postpartum women in India are multifactorial; low hemoglobin alone is not be a useful marker of iron deficiency.

## Graphical abstract

Profile of anaemia in antenatal women in India with uncomplicated pregnancies.

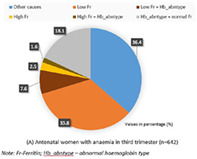

## 1 INTRODUCTION

An estimated 36% of all pregnant women, globally, have anaemia^1^ and the burden is higher in low‐and‐middle‐income countries (LMICs), particularly in India and in West and Central Africa.^1^ Anemia is “a condition in which the number and size of red blood cells, or the hemoglobin concentration, falls below an established cut‐off value.^2^” It can be caused by multiple factors such as nutritional deficiencies of iron, folate and vitamins B12, A and C, infections, chronic inflammation, and hemoglobinopathies, but the major cause of anemia in pregnancy in LMICs is thought to be iron deficiency (~50% of all anemias).^2^ While anemia is a consequence of iron deficiency, there is limited evidence about the prevalence of iron deficiency anemia (IDA) in pregnant and postpartum women.^3^ This is due to a lack of availability of low‐cost and reliable tests for biomarkers of iron status.^4^ Consequently, there is a reliance on hemoglobin alone to diagnose IDA, and to direct the use of iron to prevent and treat anemia.

India is one among 10 countries where the prevalence of anemia among pregnant women is estimated to be >50%, and has remained unchanged since 2000.^1^ This is despite free routine iron‐folic acid (IFA) supplementation (includes 60 mg elemental iron), recommended to all pregnant women for 180 days starting at second trimester of pregnancy (after 12 weeks of gestation) and for 180 days in the postnatal period.^5^, ^6^ Therefore, it is important to understand the profile of anemia and the relationship between anemia and iron status in pregnant and postpartum women in India. Focusing on two cohorts of uncomplicated pregnancies, the study objectives were to examine the relationship between anemia, of different levels of severity, and ferritin levels in antenatal and postnatal women, and soluble transferrin receptor (sTfR), transferrin saturation (TSAT), and serum hepcidin in postnatal women. Using sTfR, TSAT, and hepcidin in addition to ferritin would allow us to examine the relationship of hemoglobin with multiple iron biomarkers.

## 2 METHODS

### 2.1 Study design

We conducted a secondary analysis using data from two Indian study cohorts of the Maternal and perinatal Health Research collaboration, India (MaatHRI).^7^ Both data sets were from a population in northern India covered by 10 MaatHRI collaborating hospitals. The first data set, referred to as antenatal cohort, is from a hospital‐based prospective study, carried out between October 2018 and May 2019, that included measurements of hemoglobin and serum ferritin from 1342 pregnant women in their third trimester.^8^ The second data set is from an ongoing MaatHRI case–control study of acute cardiac complications in pregnant and postpartum women recruited between February 2018 and April 2023.^9^ This data set, referred to as the postnatal cohort, includes 909 controls of early postpartum women without any clinical history of cardiac complications recruited 12–72 h after childbirth in whom a range of iron biomarkers and hemoglobin were measured.

### 2.2 Inclusion and exclusion criteria

We included all singleton pregnancies from both data sets where hemoglobin measurements were available. To ensure that obstetric and medical complications did not bias the study results through influence on the blood parameters, women with antepartum hemorrhage, pre‐eclampsia, eclampsia, sepsis, diabetes mellitus, cardiac disease, HIV, malaria or other infections, and kidney disease were excluded from the antenatal data set as were women who received intravenous iron and/or a blood transfusion. In addition, women with postpartum hemorrhage or who had received intravenous iron and/ or blood transfusion <4 weeks prior to the blood measurements in the postnatal data set were also excluded, but not if they received iron and or a transfusion in the antenatal period >4 weeks prior to childbirth.

### 2.3 Blood parameters

Hemoglobin and serum ferritin measures were available in the antenatal data set, and data on hemoglobin, ferritin, sTfR, hepcidin, and TSAT were available in the postnatal data set. Serum ferritin is a measure of body iron‐stores and TSAT indicates iron availability to tissues, sTfR is an indicator of iron availability to the bone marrow for erythropoiesis,^4^ and hepcidin is a key iron‐regulatory hormone that controls serum iron levels.^10^ Presence of hemoglobin variants was assessed in both data sets using hemoglobin electrophoresis, and based on the results participants were grouped into normal or abnormal hemoglobin types.

As described in a previous paper,^8^ blood collection, processing, storage, and analysis are standardized for all MaatHRI studies and all blood samples were analyzed at a national laboratory in India. In both data sets, hemoglobin was measured using photometry (D×H‐800; Beckman Coulter), serum ferritin by Chemiluminescence Immunoassay (Siemens ADVIA Centaur), and High‐Performance Liquid Chromatography (Variant II Hemoglobin testing system; Bio‐Rad) was used for hemoglobin electrophoresis. Percentage of TSAT was calculated from measurements of serum iron and total iron binding capacity (measured by Spectrophotometry; Siemens Atellica), sTfR levels were measured by Nephelometry (BN II System), and hepcidin was measured using ELISA (EIA). In addition, C‐reactive protein (CRP), measured by Immunoturbidimetry (Siemens Atellica) was used to adjust ferritin levels for inflammation. Serum vitamin B12 measurements (Chemiluminescent Immunoassay, Siemens ADVIA Centaur) were available in the postnatal data set, and women with levels <200 ng/L or <148 pmol/L were classified as vitamin B12 deficient.^11^

### 2.4 Study outcomes of interest

The outcome variables were hemoglobin and anemia. Levels of severity of anemia were defined using the World Health Organization (WHO) definition for hemoglobin cut‐offs^12^: mild anemia, hemoglobin 10–10.9 g/dL, moderate 7–9.9 g/dL, and severe <7 g/dL.

### 2.5 Iron status

Iron deficiency was primarily defined using serum ferritin levels, which in the context of LMICs, is recommended as the best available measure of iron deficiency by the WHO.^4^ Postnatal ferritin measurements were adjusted for inflammation using CRP (see Section 2.6), but the antenatal measurements could not be adjusted as CRP or other markers of inflammation were not available in the antenatal data set. As there is no agreement on the physiological cut‐off values for ferritin in pregnancy and postpartum,^4^ two different cut‐off levels were used to classify women into low, normal, and high ferritin. In the antenatal cohort the usual clinical cut‐offs of <15 μg/L, 15–200 μg/L, and >200 μg/L were used for low, normal, and high, respectively^4^ as serum ferritin concentrations are lowest at the third trimester of pregnancy.^10^ Inflammation during labor and childbirth increases the ferritin levels postpartum.^10^ Therefore for the postnatal cohort, cut‐offs of <30 μg/L, 30–200 μg/L, and >200 μg/L were used, which are suggested clinical cut‐offs in pregnancy in the presence of inflammation and have been shown to have 95% sensitivity and 85% specificity in diagnosing iron deficiency in pregnant women.^13^, ^14^ sTfR is not influenced by inflammation or pregnancy^10^ and has been found to be more reliable than ferritin to measure iron deficiency in the presence of inflammation in the non‐pregnant population,^15^ but there is no agreed physiological cut‐off for this biomarker. Therefore as recommended by the WHO,^16^ we used the assay manufacturer guidance to classify postpartum women with sTfR >1.8 mg/L as iron deficient. TSAT was classified as <16%, 16%–50%, and >50% to respectively denote low, normal, and high levels in the postnatal study population.^17^ Serum hepcidin was used as a continuous variable as levels are influenced by gestational stage, lowest being at 3rd trimester and highest at first trimester and around 24 h postpartum^10^ and there are no recommended physiological cut‐offs.

### 2.6 Statistical analysis

The antenatal and postnatal data sets were analyzed separately. Based on their distribution, continuous values of the blood parameters were summarized as means with standard deviation (SD) or medians with inter‐quartile range (IQR), and proportions of women with anemia, iron status based on the four iron parameters and hemoglobin variants were calculated. We calculated the mean age and body mass index (BMI) for women in each data set. Mean gestational age at blood measurement was assessed in the antenatal data and mean hours after childbirth at blood measurement was assessed in the postnatal data set. Other population characteristics such as religion, below poverty line status, parity, gravidity, and IFA supplementation during pregnancy are reported for both cohorts. In addition, information about mean gestational age at childbirth and mode of childbirth is reported for the postnatal cohort.

Regression correction equation was used to adjust serum ferritin concentrations based on measurements of CRP.^18^ Ferritin and CRP were log transformed to achieve a normal distribution for calculating the regression coefficient for the correction equation. To avoid over‐correction, the following equation was applied only to women who had CRP levels greater than the median value of 10 mg/L^18^ in the study population.

$${\text{In Ferritin}}_{\mathrm{adj}}={\text{In Ferritin}}_{\mathrm{obs}}-\left({\beta_{1}}^{*}\left(\text{In}\;{\mathrm{CRP}}_{\mathrm{obs}}–\text{In}\;{\mathrm{CRP}}_{\mathrm{ref}}\right)\right),$$

where *β*_1_ is the regression coefficient, CRP_obs_ is the log of the original CRP measurements available in the data set, and ln CRP_ref_ is the lowest decile of In CRP_obs_).^18^

As hemoglobin was expected to be normally distributed, multivariable linear regression was used to examine the individual associations of hemoglobin with ferritin in both data sets, and with sTfR, TSAT, and hepcidin in postnatal women. The linear regression model for the antenatal cohort was adjusted for woman's age, BMI, gestational age at blood measurement and study hospital. The postnatal model was adjusted for woman's age, BMI, mode of childbirth, blood loss during childbirth, hours after childbirth at blood measurement, gestational age at childbirth, study hospital, and inflammation using CRP. The results are presented as crude and adjusted regression coefficients that ranges from +1 through 0 to −1 with 95% confidence intervals (95% CI).

Multinomial logistic regression was used to examine the association between anemia and iron status as well as anemia and having hemoglobin variants and vitamin B12 deficiency. Due to small numbers in the TSAT sub‐groups, women with moderate and severe anemia were grouped together for the TSAT analysis. The antenatal and postnatal models were adjusted for the variables mentioned above and results are presented as crude and adjusted relative risk ratio (RRR) with 95% CI, the baseline outcome group being “no anemia.” As women in the postnatal data set who received intravenous iron and/ or blood transfusion in the antenatal period >4 weeks prior to childbirth were not excluded, we conducted a sensitivity analysis excluding them to examine whether this had any material effect on the RRR.

In each model, we assessed the interaction between “hemoglobin variants” and the iron biomarkers, and if a significant interaction was found, models were re‐run stratified by the interacting variable. Missing data for the blood parameters were expected because of samples being discarded due to quality issues, but was not related to the measurements of the blood biomarkers. Therefore, data in the study were considered to be missing at random and complete case analysis was conducted. All statistical tests were considered significant if two‐tailed *p*‐value was <.05. Analyses were undertaken using Stata V.17.0, MP‐Parallel Edition (StataCorp).

## 3 RESULTS

### 3.1 Study population

Based on inclusion and exclusion criteria, 1132 out of 1342 pregnant women were eligible for the antenatal analysis, and 837 out of 909 postpartum women were eligible for the postnatal analysis. A flow diagram of the selection of the study participants is included in Figure S1. Mean age of the women included in each study cohort was 25 years and their BMI was also comparable, 21 kg/m^2^ in the antenatal and 22 kg/m^2^ in the postnatal cohort (Table S1). Mean gestational age at blood collection was 35 (SD 0.11) weeks in the antenatal cohort. In the postnatal cohort, the mean gestational age at childbirth was 38.4 weeks (SD 2.4) and on an average, the blood samples were collected at 25.4 (SD 0.72) hours from childbirth. Around 94% of antenatal and 98% of postnatal women received routine IFA supplementation during pregnancy and 69% of pregnant and 72% of postpartum women reported to have consumed IFA tablets for ≥100 days during pregnancy. A majority of women were Hindus in both cohorts. A higher proportion of women in the postnatal cohort were below the poverty line (62%) compared with 47% in the antenatal cohort. A majority of the study population in both cohorts were nulliparous and primigravida. A majority of the women in the postnatal cohort had spontaneous vaginal birth (69%). Hemoglobin was found to be normally distributed and the mean hemoglobin concentration was comparable in the two data sets (~10.5 g/dL). Median serum ferritin levels were higher in the postnatal cohort (47.5 μg/L) compared with the antenatal cohort (24.7 μg/L), despite postnatal measurements being corrected for inflammation. Median values of the other iron biomarkers, available only for the postnatal cohort, are shown in Table S1.

The prevalence of moderate anemia (32% in antenatal; 31% in postnatal cohort) and severe anemia (3% in antenatal; 4% in postnatal cohort) were similar in the two cohorts, although there was a higher prevalence of mild anemia in the postnatal cohort (26% in postnatal; 21% in antenatal). The proportion of women with low (37%) and high ferritin (6%), corrected for inflammation, was higher in the postnatal cohort compared with 31% low and 4% high ferritin in the antenatal cohort (Table S1). Data available on other iron biomarkers in the postnatal cohort reported low iron availability to the bone marrow (sTfR > 1.8 mg/L) in 30%, low circulating iron (TSAT < 16%) in 55%, and high TSAT (>50%) in 8% of the study population. Descriptive statistics comparing iron status by severity of anemia in the two study cohorts are presented in Tables S2 and S3. Importantly, we observed that even among women with no anemia, in the antenatal cohort 17% had low ferritin, and in the postnatal cohort 27% had low ferritin, 17% had high sTfR and 57% had low TSAT.

**FIGURE 1:**
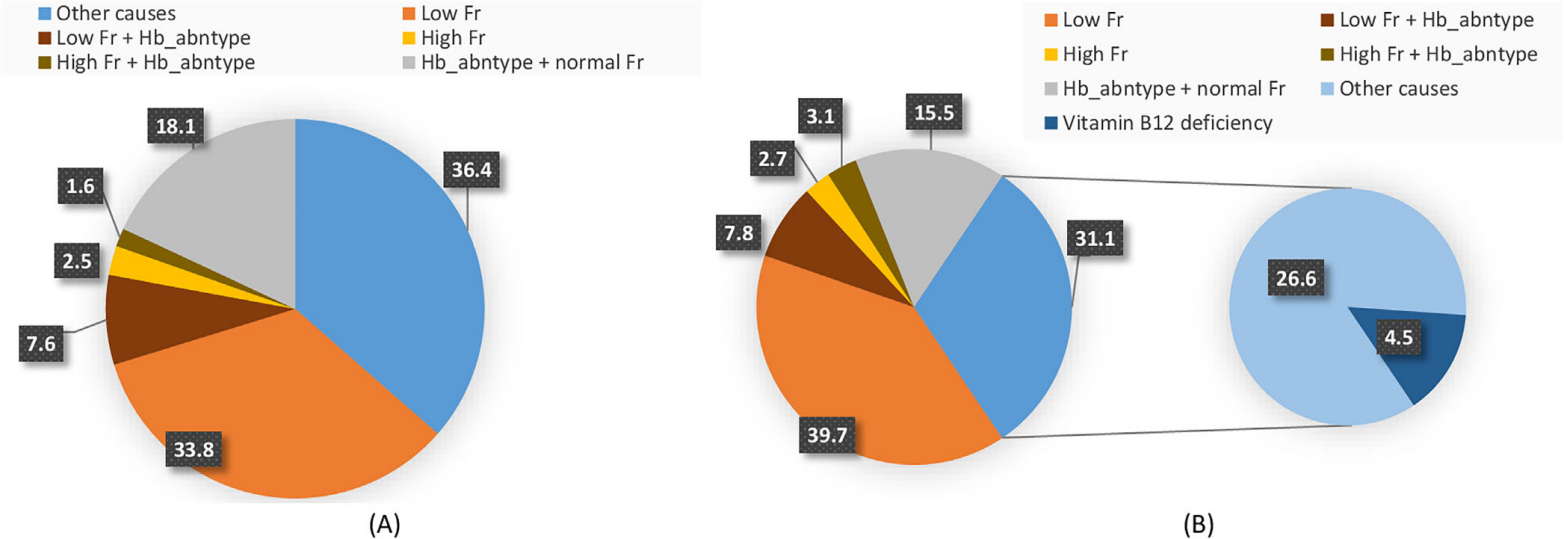
Profile of anemia in antenatal and postnatal women in India with uncomplicated pregnancies. (A) Antenatal women with anemia in third trimester (*n* = 642); values shown are in percentage (%). (B) Postnatal women with anemia at 12–72 h after childbirth (*n* = 511); values shown are in percentage (%). Fr, ferritin; Hb_abntype, abnormal hemoglobin type. [Color figure can be viewed at wileyonlinelibrary.com]

The profile of anemia in this study population with uncomplicated pregnancies is shown in Figure 1A,B. Among women who had anemia, around 34% of the antenatal and 40% of the postnatal cohort had low ferritin, 8% in both cohorts had a combination of low ferritin and abnormal hemoglobin types, 4% antenatal and 6% postnatal women had high ferritin or high ferritin with abnormal hemoglobin types, another 18% antenatal and 16% postnatal women had abnormal hemoglobin types with normal ferritin levels, and the remaining 36% and 31% of antenatal and postnatal women, respectively had other causes of anemia. Among the postnatal anemic women, 4.5% of the other causes were vitamin B12 deficiency (Figure 1B), but B12 deficiency was also found to co‐exist with the other causes in the study population.

Around 22% of all women in each study cohort had abnormal hemoglobin types (Table S1), and its likelihood was significantly higher for the mild (antenatal: adjusted relative risk ratio (aRRR) 2.10, 95% CI 1.42, 3.09; postnatal: aRRR 1.89, 95% CI 1.17, 3.08) and moderate anemia (antenatal: aRRR 2.30, 95% CI 1.63, 3.25; postnatal: aRRR 3.03, 95% CI 1.94, 4.73) groups compared with “no anemia” (Table S4), but not for women with severe anemia (antenatal: aRRR 1.01, 95% CI 0.40, 2.54; postnatal: aRRR 1.79, 95% CI 0.61, 5.23). The most common hemoglobin variant was HbE; prevalence of trait was 9% in the antenatal cohort and 13% in the postnatal cohort, and another 4% women in each cohort were homozygous. This was followed by β‐thalassemia trait, around 5% in the antenatal and 2% in the postnatal cohort; and 0.3% in the antenatal 0.5% in the postnatal cohort had sickle cell trait. The remaining proportions of the variants were unspecified. We also found the prevalence of vitamin B12 deficiency to increase with the severity of anemia in the postnatal cohort (Figure S2), but the risk of deficiency was significantly higher only in the moderate anemia group compared with women with no anemia (aRRR 1.75, 95% CI 1.18, 2.61) after adjusting for potential confounders (Table S4).

### 3.2 Relationship between hemoglobin and iron biomarkers

**FIGURE 2:**
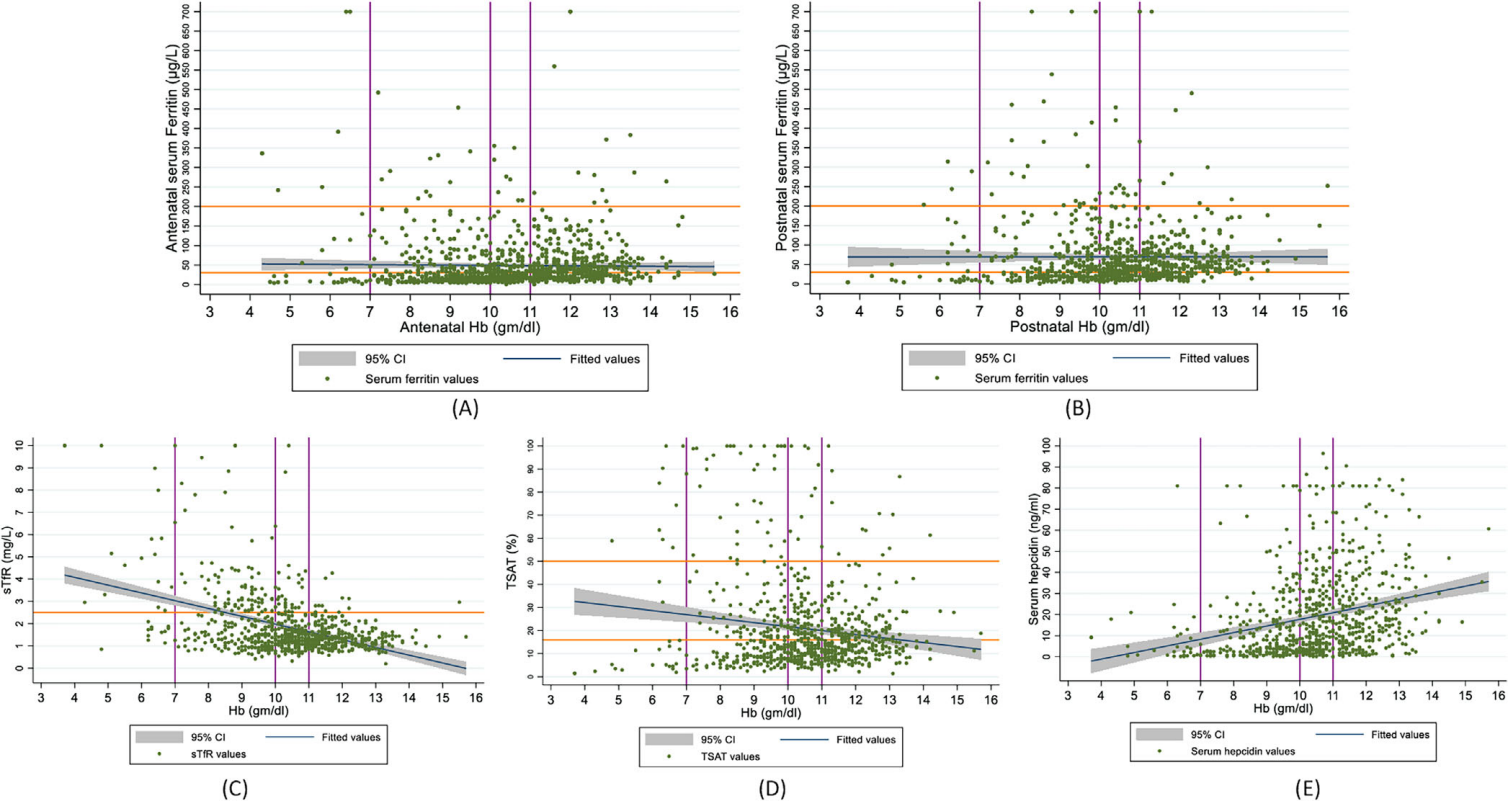
Relationship between hemoglobin and iron biomarkers in antenatal and postnatal women in India with uncomplicated pregnancies. (A) Antenatal ferritin (showing cut‐offs for anemia and ferritin levels). (B) Postnatal ferritin (showing cut‐offs for anemia and ferritin levels). (C) Postnatal sTfR (showing cut‐offs for anemia and sTfR). (D) Postnatal TSAT (showing cut‐offs for anemia and TSAT). (E) Postnatal hepcidin (showing cut‐offs for anemia). [Color figure can be viewed at wileyonlinelibrary.com]

We did not find evidence of a relationship between hemoglobin and serum ferritin in the study cohorts (Figure 2A,B) and the relationship did not change after adjusting for potential confounders (Table 1). The magnitudes of association were also very similar in the two cohorts (antenatal: adjusted coefficient [aCoef] −0.0004, 95% CI −0.001, 0.001; postnatal: aCoef −0.0001, 95% CI −0.001, 0.001). There was evidence of interaction between “hemoglobin variants” and ferritin in their association with hemoglobin levels (interaction test *p*‐value .012), but the stratified results did not vary substantially between the groups of normal and abnormal types (Table S5).

**TABLE 1:** Relationship between hemoglobin and iron biomarkers in women with uncomplicated pregnancies.

Iron biomarkers	Antenatal Hb				Postnatal Hb			
	Cr Coef (95% CI)	*p*‐value	Adj Coef^a^ (95% CI)	*p*‐value	Cr Coef^b^ (95% CI)	*p*‐value	Adj Coef^c^ (95% CI)	*p*‐value
Serum ferritin in μg/L	−0.0003 (−0.001 to 0.001)	.645	−0.0004 (−0.001 to 0.001)	.489	−0.0004 (−0.002 to 0.001)	.519	−0.0001 (−0.001 to 0.001)	.801
sTfR in mg/L	–	–	–	–	−0.07 (−0.09 to −0.04)	<.001	−0.04 (−0.07 to −0.01)	.003
TSAT in %	–	–	–	–	−0.005 (−0.008 to −0.002)	.001	−0.005 (−0.008 to −0.002)	.002
Serum hepcidin in ng/mL	–	–	–	–	0.03 (0.02–0.03)	<.001	0.02 (0.02–0.03)	<.001

^a^ Adjusted for woman's age and BMI, gestational age at blood measurement, study hospital.

^b^ Adjusted for inflammation using C‐reactive protein.

^c^ Adjusted for woman's age, BMI, mode of childbirth, blood loss during childbirth, hours after childbirth at blood measurement, gestational age at childbirth, study hospital, and inflammation using C‐reactive protein.

Although we found a significant negative linear association of hemoglobin with sTfR (aCoef −0.04, 95% CI −0.07, −0.01) and TSAT (aCoef −0.005, 95% CI −0.008, −0.002), and a positive linear association with hepcidin (aCoef 0.02, 95% CI 0.02, 0.03) in the postnatal cohort, the coefficients of association were small (Table 1) and did not change after adjusting for potential confounders or by excluding women who had a history of receiving intravenous iron/blood transfusion >4 weeks prior to blood sample collection. Figure 2C–E show the relationships of hemoglobin with sTfR, TSAT, and hepcidin, respectively. Upon stratification by “hemoglobin variants,” the magnitudes of the association between postnatal hemoglobin and two iron biomarkers, namely ferritin and sTfR were varied (ferritin: normal hemoglobin type aCoef 0.001, 95% CI −0.001, 0.002; abnormal type aCoef −0.002, 95% CI −0.004, −0.0002; sTfR: normal type aCoef −0.03, 95% CI −0.06, −0.01; abnormal type aCoef −0.44, 95% CI −0.63, −0.25), but did not vary substantially for hepcidin (normal type aCoef 0.03, 95% CI 0.02, 0.04; abnormal type aCoef 0.01, 95% CI 0.003, 0.02). Overall, the negative linear associations of hemoglobin with ferritin and sTfR were accentuated in the group with abnormal hemoglobin types (Table S5). There was no evidence of interaction between “hemoglobin variants” and TSAT in their association with hemoglobin.

### 3.3 Relationship between anemia and ferritin levels in antenatal and postnatal women

In both cohorts, the risk of low ferritin was higher across all levels of severity of anemia (Table 2). After adjusting for potential confounders, in the antenatal cohort, compared with women with no anemia, the risk of low ferritin was more than two‐fold higher in women with mild anemia (aRRR 2.36, 95% CI 1.62, 3.42), more than four‐fold higher in moderate (aRRR 4.37, 95% CI 3.15, 6.07) and nearly 14‐fold higher in severe anemia (aRRR 13.8, 95% CI 5.95, 31.9). In the postnatal cohort this risk was 66% (aRRR 1.66, 95% CI 1.11, 2.47), nearly three‐fold (aRRR 2.56, 95% CI 1.76, 3.72), and more than three‐fold (aRRR 3.19, 95% CI 1.17, 8.71) higher in women with mild, moderate and severe anemia, respectively. Compared with non‐anemic women, the risk of high ferritin was found to be significantly higher only among antenatal women with severe anemia (aRRR 28.6, 95% CI 8.84, 92.7), and among postnatal women with moderate (aRRR 2.36, 95% CI 1.02, 5.43) and severe anemia (aRRR 7.14, 95% CI 1.54, 33.1). Upon stratification by “hemoglobin variants,” magnitudes of the association between antenatal anemia and ferritin levels did not vary substantially between the groups of normal or abnormal hemoglobin types (Table S6). In the postnatal cohort, upon stratification, magnitude of the association between postnatal anemia and low ferritin was accentuated among women with normal hemoglobin types (mild aRRR 1.82, 95% CI 1.17, 2.85; moderate aRRR 3.62, 95% CI 2.34, 5.60; severe anemia aRRR 5.23, 95% CI 1.61, 17.04), but the aRRR for high ferritin was significantly attenuated across all categories of anemia (Table S7). The opposite effect was observed among women with abnormal hemoglobin types, where the aRRR for low ferritin was attenuated across all anemia categories, but the aRRR was accentuated for high ferritin in the moderate (aRRR 6.31, 95% CI 0.70, 56.7) and severe anemia groups (aRRR 58.6, 95% CI 1.94, 1777.3). However, due to small numbers, the 95% CI was much wider for all aRRRs and the results should be interpreted with caution (Table S7).

**TABLE 2:** Relationship between anemia and iron biomarkers in women with uncomplicated pregnancies.

Serum ferritin								
No anemia	Baseline outcome				Baseline outcome			
Anemia	Antenatal				Postnatal			
	cRRR (95% CI)	*p*‐value	aRRR^a^ (95% CI)	*p*‐value	cRRR^b^ (95% CI)	*p*‐value	aRRR^c^ (95% CI)	*p*‐value
Mild anemia								
Low iron‐stores	2.31 (1.61–3.31)	<.001	2.36 (1.62–3.42)	<.001	1.69 (1.16–2.44)	.006	1.66 (1.11–2.47)	.013
Normal iron‐stores	1 (Ref)		1 (Ref)		1 (Ref)		1 (Ref)	
High iron‐stores	1.57 (0.64–3.85)	.329	1.84 (0.74–4.58)	.190	1.08 (0.41–2.82)	.878	0.61 (0.19–1.98)	.410
Moderate anemia								
Low iron‐stores	4.14 (3.03–5.67)	<.001	4.37 (3.15–6.07)	<.001	2.51 (1.76–3.58)	<.001	2.56 (1.76–3.72)	<.001
Normal iron‐stores	1 (Ref)		1 (Ref)		1 (Ref)		1 (Ref)	
High iron‐stores	1.77 (0.78–4.03)	.172	2.06 (0.89–4.73)	.090	2.72 (1.25–5.92)	.011	2.36 (1.02–5.43)	.044
Severe anemia								
Low iron‐stores	10.8 (4.76–24.3)	<.001	13.8 (5.95–31.9)	<.001	4.62 (1.85–11.5)	.001	3.19 (1.17–8.71)	.023
Normal iron‐stores	1 (Ref)		1 (Ref)		1 (Ref)		1 (Ref)	
High iron‐stores	23.5 (7.56–72.7)	<.001	28.6 (8.84–92.7)	<.001	9.85 (2.52–38.5)	.001	7.14 (1.54–33.1)	.012

Other iron biomarkers (sTfR and TSAT)								
No anemia	—	—	—	—	Baseline outcome			
Mild anemia	—	—	—	—				
sTfR ≤ 1.8 mg/L	—	—	—	—	1 (Ref)		1 (Ref)	
sTfR > 1.8 mg/L	—	—	—	—	1.74 (1.14–2.65)	.010	1.66 (1.05–2.62)	.029
Moderate anemia	—	—	—	—				
sTfR ≤ 1.8 mg/L	—	—	—	—	1 (Ref)		1 (Ref)	
sTfR > 1.8 mg/L	—	—	—	—	4.23 (2.88–6.20)	<.001	3.98 (2.65–5.97)	<.001
Severe anemia	—	—	—	—				
sTfR ≤ 1.8 mg/L	—	—	—	—	1 (Ref)		1 (Ref)	
sTfR > 1.8 mg/L	—	—	—	—	10.6 (4.57–24.5)	<.001	6.69 (2.54–17.6)	<.001

No anemia	—	—	—	—	Baseline outcome			
Mild anemia	—	—	—	—				
TSAT < 16%	—	—	—	—	0.89 (0.62–1.29)	.548	0.91 (0.61–1.35)	.642
TSAT 16%–50%	—	—	—	—	1 (Ref)		1 (Ref)	
TSAT > 50%	—	—	—	—	0.99 (0.40–2.42)	.983	0.82 (0.31–2.17)	.686
Moderate/severe anemia	—	—	—	—				
TSAT < 16%	—	—	—	—	1.14 (0.80–1.63)	.454	1.02 (0.70–1.49)	.912
TSAT 16%–50%	—	—	—	—	1 (Ref)		1 (Ref)	
TSAT > 50%	—	—	—	—	4.67 (2.37–9.22)	<.001	3.91 (1.94–7.90)	<.001

*Note*: Antenatal: low iron‐stores <15 μg/L, normal 15–200 μg/L, high >200 μg/L; postnatal: low iron‐stores <30 μg/L, normal 30–200 μg/L, high >200 μg/L.

Abbreviation: RRR, relative risk ratio.

^a^ Adjusted for woman's age and BMI, gestational age at blood measurement, study hospital.

^b^ Adjusted for inflammation using C‐reactive protein.

^c^ Adjusted for woman's age, BMI, mode of childbirth, blood loss during childbirth, hours after childbirth at blood measurement, gestational age at childbirth, study hospital, and inflammation using C‐reactive protein.

On further exploration, we found that 35% of the women with high ferritin (>200 μg/L) in the antenatal cohort and 41% in the postnatal cohort had abnormal hemoglobin types, compared with around 24% in the normal and 17% in the low ferritin groups in both data sets (Figure S3A,B), suggestive of potential iron loading hemoglobinopathies leading to high ferritin among some women. However, median hepcidin levels were significantly higher in the high ferritin group (30.4 ng/mL) compared with the other two groups (normal ferritin group 16.9 ng/mL; and low ferritin group 4.7 ng/mL) in the postnatal data set (Figure S4A), reflecting appropriate hepcidin regulation by iron.

### 3.4 Relationship between anemia and other iron biomarkers in postnatal women

We found a higher risk of low iron availability to the bone marrow (sTfR > 1.8 mg/L) across all categories of anemia; women with mild, moderate, and severe anemia had almost two‐fold (aRRR 1.66, 95% CI 1.05, 2.62), four‐fold (aRRR 3.98, 95% CI 2.65, 5.97) and seven‐fold (aRRR 6.69, 95% CI 2.54, 17.6) higher risk of low iron availability, respectively, compared with women with no anemia (Table 2). We did not find the likelihood of low TSAT to differ between anemic and non‐anemic women, however women with moderate–severe anemia had nearly four‐fold increased risk of high TSAT levels (>50%) compared with women with no anemia (aRRR 3.91, 95% CI 1.94, 7.90). Similar to high ferritin, possible reasons for high TSAT were explored by examining the associations of TSAT with hemoglobin variants and hepcidin. In contrast to ferritin, we did not find TSAT to vary significantly across the groups of normal and abnormal hemoglobin types (*p* = .240; Figure S5). However, we found a significant interaction between hepcidin and TSAT (*p* < .001). Median hepcidin levels were lowest among postnatal women with TSAT > 50% (8.7 ng/mL), compared with 11.3 ng/mL in the low TSAT and 15.8 ng/mL in the normal TSAT groups (Figure S4B).

Upon stratification by “hemoglobin variants,” magnitudes of the associations did not materially vary for sTfR and TSAT in the groups with normal and abnormal hemoglobin types across the categories of anemia. Association between postnatal anemia and iron biomarkers, including for high ferritin and high TSAT, did not change after excluding women who received intravenous iron and/or blood transfusion >4 weeks prior to blood collection.

## 4 DISCUSSION

The study showed that despite a high proportion of antenatal and postnatal women receiving IFA supplementation, 57% of antenatal and 61% of postnatal women with uncomplicated pregnancies were anemic in the study population. Around a third of the women had low ferritin, but 4% women in the antenatal and 6% in the postnatal cohort also had high ferritin levels. Overall, hemoglobin was poorly associated with all four iron biomarkers, ferritin, sTfR, TSAT, and hepcidin, indicating that low hemoglobin alone is not a useful marker of iron deficiency in pregnant and postpartum women. However, women across all severity groups of anemia (mild, moderate, and severe) had low ferritin. A high likelihood of iron deficiency across all severities of anemia in postnatal women was also shown by high levels of sTfR, a biomarker not considered to be influenced by inflammation or pregnancy/postpartum status. However, we cannot ignore that some women with moderate–severe anemia could also have a risk of iron‐overload due to high ferritin and high TSAT levels, respectively mediated by high and low hepcidin levels, a protein that regulates iron absorption and mobilization from body stores. Furthermore, women with high ferritin levels also had a higher likelihood of having abnormal hemoglobin types. Again importantly, our study showed that 17%–57% (based on the iron biomarker used) of non‐anemic women had iron deficiency. Thus the study findings suggest that in the Indian population, the causes of anemia are multifactorial and the relationship between anemia and iron status in pregnancy and postpartum is complex.

In India^6^ and other LMICs, low hemoglobin in pregnancy is used as an indication for iron supplementation and treatment, although anemia and iron deficiency do not completely overlap.^19^ Our study demonstrates this and shows that low hemoglobin is not a useful indicator of iron deficiency anemia. There is a need to measure iron biomarkers to ascertain the need for supplementation and treatment. Non‐anemic iron deficiency has been shown to be associated with adverse outcomes in non‐pregnant populations.^15^ If based on hemoglobin measurement only, pregnant women who are iron deficient but not anemic are not treated for iron deficiency, this would put them at greater risk of developing anemia later and could increase the risk of adverse maternal and fetal outcomes. Conversely, others who are anemic but not iron deficient are being unnecessarily supplemented, putting them at risk of being exposed to excess iron. Ineffective erythropoiesis is observed in hemoglobinopathies such as thalassemia, hereditary sideroblastic anemias, and certain myelodysplastic syndromes, in which anemia is associated with iron overload, the latter resulting from increased iron absorption and mobilization of iron from the stores mediated by relative hepcidin deficiency.^20^, ^21^ A significant proportion of our study population with high ferritin had abnormal hemoglobin types (35% in the antenatal and 41% in the postnatal cohort). Therefore, these conditions should be investigated before treating women with iron assuming that the observed anemia is due to iron deficiency.

Hepcidin levels are known to increase dramatically around the first 24 h postpartum,^10^ but in some postpartum women with moderate–severe anemia in our study population, hepcidin levels were inappropriately low which was associated with high TSAT. In these women, low hepcidin might have led to continued increased mobilization of iron into the circulation from the iron‐stores but there was no further transfer of iron from the maternal to the fetal circulation after childbirth. Several factors other than iron could influence hepcidin levels including inflammation, hypoxia, liver disease, obesity, and erythropoietic and hormonal factors,^21^, ^22^ although we mostly excluded women with medical conditions that could be associated with these factors. Because we did not have TSAT measurements in the antenatal cohort, we could not assess the association between anemia and TSAT in the antenatal period when a considerable proportion of maternal circulating iron is transferred to the feto‐placental unit.

In the postnatal cohort, women with moderate anemia were found to have a higher risk of vitamin B12 deficiency compared with non‐anemic women. This is not surprising as most nutritional deficiencies co‐exist, so it is important to treat B12 deficiency in addition to iron deficiency to reduce the burden of anemia in pregnant and postpartum women in India. Although there is no consensus on an ideal biomarker for vitamin B12 deficiency in pregnancy and early postpartum,^11^ an individual data meta‐analysis showed a significant association of low maternal serum vitamin B12 levels (<148 pmol/L) with preterm birth and low birthweight,^23^ and supplementation for pregnant women is recommended in areas where B12 deficiency has a high prevalence.^11^ Our results are also supported by studies from India that show that anemia due to vitamin B12 deficiency is endemic in the country due to high prevalence of vegetarianism^24^ including among pregnant women.^25^, ^26^

Despite implementation of national programs for routine iron supplementation for pregnant and postpartum women since 1970,^5^, ^27^ anemia in pregnancy is still a major problem in India and data from the fifth round of the National Family Health Survey (NFHS‐5) showed an increase in prevalence of anemia (52.2%) in pregnant women compared with NFHS‐4 (50.4%).^28^ These figures are comparable to the prevalence in our study population. Non‐adherence with regular supplementation is thought to be a major reason for the lack of success of the iron supplementation programmes,^29^, ^30^ but we found that despite high reported consumption of IFA, the prevalence of anemia was more than 55% and that the causes were multifactorial with iron deficiency being the cause in just over a third of the anemic women. Furthermore, the observations in our study of a minimal association between hemoglobin and iron biomarkers could explain why trials based on hemoglobin alone as a marker of iron deficiency anemia for assessment of outcome of supplementation are not effective. For example, a recent trial by Pasricha et al.^31^ could not show differences in hemoglobin as a primary outcome when comparing anemic pregnant women receiving intravenous iron with those receiving oral iron, although a significant correction of iron deficiency and overall reduction of iron deficiency anemia was noted in the group receiving intravenous iron.

Ours is the first study to examine in detail the association between anemia and iron parameters in both antenatal and postnatal women in an LMIC setting. Among strengths of our study is its focus on multiple markers of iron status from a large number of participants with uncomplicated pregnancies. Although the data sets included two separate population cohorts, their descriptive characteristics, including prevalence of anemia, and findings relating to ferritin and hemoglobin variants were comparable. Furthermore, study data in both data sets were collected using the same data collection tools, the same laboratory and assay methods were used for biomarkers, and the population were from the same geographical areas covered by the 10 MaatHRI collaborating hospitals. Nevertheless, the limitations included smaller numbers in the severe anemia sub‐group especially in the postnatal data set, and the lack of iron biomarkers beyond ferritin in the antenatal cohort.

Biomarker measurements were missing for several participants in both data sets, which could have biased the results, although the likelihood of such bias is low as the data were missing at random. We were unable to adjust ferritin levels for inflammation in the antenatal cohort and were able to use only one inflammatory marker (CRP) for adjusting ferritin levels in the postnatal cohort, the recommendation is to use two inflammatory markers.^4^ We did not find any reports of worm infestation in the medical history of the study participants, which is an important cause of iron deficiency and inflammation, and therefore could not account for this in the analysis. This might have resulted in under‐correction for inflammation, although the prevalence of low and high ferritin levels were comparable in the two cohorts and our study included women with uncomplicated pregnancies. As the blood samples were not collected after an overnight fasting, iron biomarkers like TSAT could be influenced by diurnal variation as well as food intake, but these effects are likely to be random and would not lead to a systematic bias. However, the British Society of Hematology do not recommend overnight fasting for measurement of TSAT,^32^, ^33^ but suggests that borderline results could be repeated or checked on fasting samples, if desired.^32^

Again, although we excluded women with obstetric and medical complications, the results could still be influenced by unmeasured confounding. Due to a lack of clinical diagnosis, we were unable to confirm the presence and types of hemoglobinopathies, but the most common hemoglobin variant identified through hemoglobin electrophoresis was HbE trait followed by HbE homozygous, β‐thalassemia trait and sickle cell trait. The findings of the study may not be completely generalizable to populations in which the prevalence of anemia, iron deficiency, and hemoglobin variants are different from our study population in India, but we expect the physiological relationship between hemoglobin and the iron parameters to be comparable.

## 5 CONCLUSION

The relationship between anemia and iron status in pregnant and postpartum women in India is complex and the causes of anemia are multifactorial. The findings demonstrate that it is important to improve the identification, measurement, and understanding of anemia to facilitate appropriately tailored prevention, control, and treatment activities in India, and not provide oral and intravenous iron on the basis of low hemoglobin alone. This evidence is aligned with the WHO's action plan for achieving the Global Nutrition Target‐2 of a 50% reduction of anemia in women of reproductive age group by 2025.^2^ It is important to remember that many non‐anemic women could be iron deficient and may need tests for iron biomarkers. On the other hand, it is important to reiterate that iron biomarkers, and not just hemoglobin, should be measured before starting treatment with oral or intravenous iron, and if a pregnant or postpartum woman with anemia is not responding to treatment, iron should be stopped and tests for presence of hemoglobinopathies and other causes of anemia should be explored. There is therefore an urgent need to develop low‐cost reliable diagnostics tests for biomarkers of iron status such as sTfR which are not influenced by inflammation or pregnancy. Furthermore, the findings imply that iron intervention trials should use iron biomarkers as primary endpoints and not hemoglobin alone, and there is a need for trials to test the effectiveness of a combination of iron and vitamin B12 compared with iron alone. More in‐depth research with repeated measurements of hemoglobin and iron biomarkers across the pregnancy and postpartum period is also needed.

## AUTHOR CONTRIBUTIONS

Manisha Nair developed the concept for the study, is the chief investigator of the MaatHRI projects from which the data sets were used, analyzed the data, and wrote the first draft of the paper. Saswati S. Choudhury, Anjali Rani, Carolin Solomi V., Swapna D. Kakoty, Robin Medhi, Sereesha Rao, Pranabika Mahanta, and Indrani Roy contributed equally. They are collaborators and investigators of the two MaatHRI projects from which the data sets were used, and contributed to the interpretation of the findings, and edited the paper. Farzana Zahir, Shakuntala Chhabra, Gitanjali Deka, and Bina Minz contributed equally in terms of being collaborators and investigators for one of the two MaatHRI studies from which the data were used. They also contributed to the interpretation of the findings, and edited the paper. Rupanjali Deka is the MaatHRI Project Manager who supervised data collection and edited the paper. Charles Opondo is a bio‐statistician and provided statistical support for the analysis and edited the paper. David Churchill contributed to the interpretation of the results and edited the paper. Samira Lakhal‐Littleton and Elizabeta Nemeth contributed to the concept, development of the analysis plan, interpretation of the results and edited the paper.

## FUNDING INFORMATION

The MaatHRI platform and this study are funded by a MRC Career Development Award to MN (Ref: MR/P022030/1) and a Transition Support Award for MN (Ref: MR/W029294/1). EN is funded by NICHD grants R21HD098864 and R01HD096863. The funders had no role in the study design, data collection, analysis, or writing the paper.

## CONFLICT OF INTEREST STATEMENT

Manisha Nair declares a MRC Transition Support Award (Ref: MR/W029294/1), including support for attending meetings and/or travel. Elizabeta Nemeth is a scientific cofounder of Intrinsic LifeSciences and Silarus Therapeutics and a consultant for Protagonist, Vifor, RallyBio, Ionis, GSK, Novo Nordisk, AstraZenecaFibrogen and Disc Medicine. David Churchill is a member of the multi‐disciplinary iron deficiency anemia steering group run by Pharmacosmos and receives an honorarium for that work.

## PATIENT CONSENT

Written informed consent was obtained from all participants prior to data and blood sample collection.

## Supporting information


**Figure S1.** Flow diagram of the selection of the study participants.
**Figure S2.** Prevalence of vitamin B12 deficiency by severity of anemia in postnatal women (12–72 h after childbirth).
**Figure S3.** Serum ferritin levels and hemoglobin variants in antenatal and postnatal women with uncomplicated pregnancies.
**Figure S4.** Iron status and median serum hepcidin in postnatal women with uncomplicated pregnancies.
**Figure S5.** TSAT levels and hemoglobin variants in postnatal women (12–72 h after childbirth).
**Table S1.** Characteristics of the study population.
**Table S2.** Descriptive statistics comparing anemia and iron status in the antenatal cohort.
**Table S3.** Descriptive statistics comparing anemia and iron status in the postnatal cohort.
**Table S4.** Relationship between anemia, hemoglobin variants, and vitamin B12 deficiency in women with uncomplicated pregnancies.
**Table S5.** Relationship between hemoglobin and iron biomarkers stratified by hemoglobin variants.
**Table S6.** Association between antenatal anemia and ferritin levels stratified by hemoglobin variants.
**Table S7.** Association between postnatal anemia and ferritin levels stratified by hemoglobin variants.

## Data Availability

Data included in the paper is available for free and can be obtained by contacting the corresponding author or by emailing data.access@ndph.ox.ac.uk.
