# Supplementary material for: The complex relationship between iron status and anemia in pregnant and postpartum women in India: Analysis of two Indian study cohorts of uncomplicated pregnancies
Source: Am J Hematol. 2023 Aug 31;98(11):1721–31. doi: 10.1002/ajh.27059 (PMC11441206; doi:10.1002/ajh.27059)
Supplement: Supplementary file 1 — Figure S1. Flow diagram of the selection of the study participants. Figure S2. Prevalence of vitamin B12 deficiency by severity of anemia in postnatal women (12–72 h after childbirth). Figure S3. Serum ferritin levels and hemoglobin variants in antenatal and postnatal women with uncomplicated pregnancies. Figure S4. Iron status and median serum hepcidin in postnatal women with uncomplicated pregnancies. Figure S5. TSAT levels and hemoglobin variants in postnatal women (12–72 h after childbirth). Table S1. Characteristics of the study population. Table S2. Descriptive statistics comparing anemia and iron status in the antenatal cohort. Table S3. Descriptive statistics comparing anemia and iron status in the postnatal cohort. Table S4. Relationship between anemia, hemoglobin variants, and vitamin B12 deficiency in women with uncomplicated pregnancies. Table S5. Relationship between hemoglobin and iron biomarkers stratified by hemoglobin variants. Table S6. Association between antenatal anemia and ferritin levels stratified by hemoglobin variants. Table S7. Association between postnatal anemia and ferritin levels stratified by hemoglobin variants. [file AJH-98-1721-s001.docx]

**Online supplementary material**


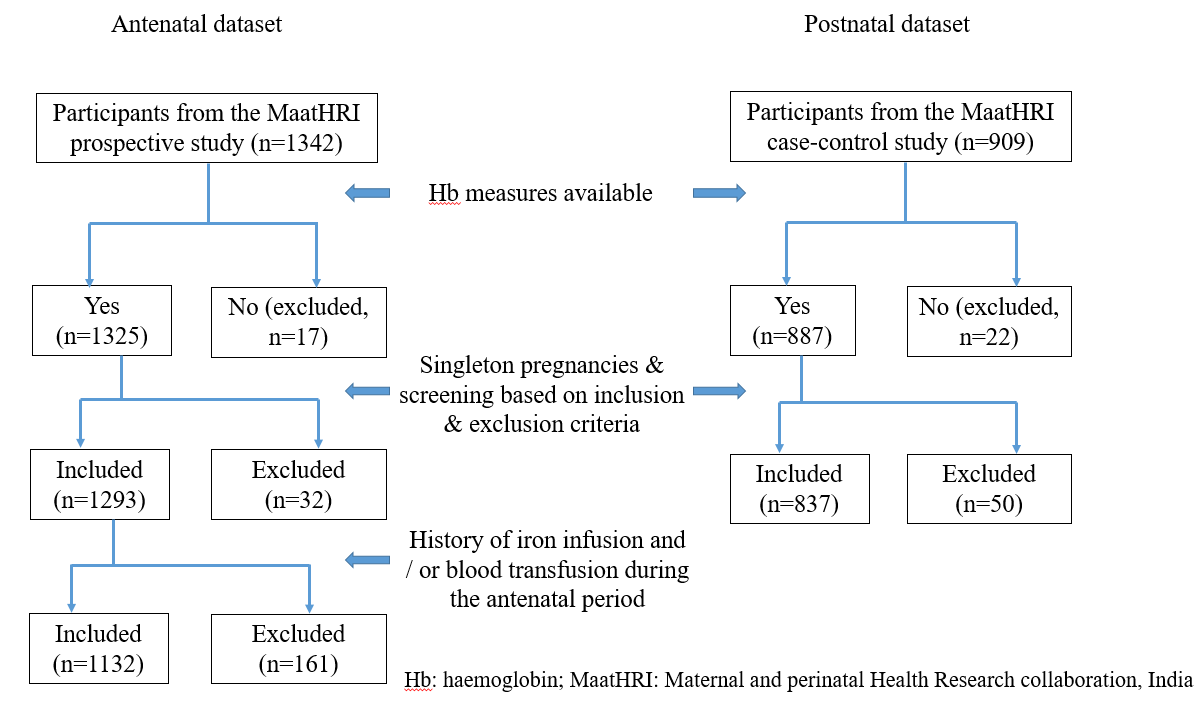


**Figure-S1: Flow diagram of the selection of the study participants**

**Figure-S2: Prevalence of vitamin B12 deficiency by severity of anaemia in postnatal women (12-72 hours after childbirth)**

(A) Antenatal study cohort (B) Postnatal study cohort

**Figure S3: Serum ferritin levels and haemoglobin variants in antenatal and postnatal women with uncomplicated pregnancies**

S4 (A)Ferritin levels and hepcidin S4 (B) TSAT levels and hepcidin

**Figure S4: Iron status and median serum hepcidin in postnatal women with uncomplicated pregnancies**

**Figure S5: TSAT levels and haemoglobin variants in postnatal women (12-72 hours after childbirth)**

**Table-S1: Characteristics of the study population**

| Participant characteristics | Antenatal study cohort (n=1132) | Postnatal study cohort (n= 837) |
| --- | --- | --- |
|  | Mean (­SD) | Mean (­SD) |
| Woman’s age (in years) | 24.5 (0.12) | 24.6 (0.17) |
| Woman’s body mass index (BMI in kg/m^2^) | 21.4 (0.10) | 22.3 (0.11) |
| Gestational age at blood measurement (in weeks) | 35.3 (0.11) | -- |
| Mean gestational age at childbirth (in weeks) | -- | 38.4 (2.4) |
| Hours since childbirth at blood measurement | -- | 25.4 (0.72) |
| Haemoglobin (in g/dL) | 10.5 (1.8) | 10.4 (1.7) |
| Anaemia | Frequency (%) | Frequency (%) |
| No anaemia (≥11g/dL) | 490 (43.3) | 326 (38.9) |
| Mild anaemia (10-10.9g/dL) | 239 (21.1) | 219 (26.2) |
| Moderate anaemia (Hb 7-9.9 g/dL) | 366 (32.3) | 261 (31.2) |
| Severe anaemia (Hb <7 g/dL) | 37 (3.3) | 31 (3.7) |
| Haemoglobin variants |  |  |
| Normal haemoglobin type | 876 (77.4) | 649 (75.5) |
| Abnormal haemoglobin types^$^ | 246 (21.7) | 180 (21.5) |
| Missing | 10 (0.9) | 8 (0.9) |
| Vitamin B12 deficiency^*^ | -- |  |
| No | -- | 510 (60.9) |
| Yes | -- | 226 (27.0) |
| Missing | -- | 101 (12.1) |
| Oral iron-folic acid supplementation |  |  |
| Not received | 71 (6.3) | 14 (1.8) |
| For <100 days | 280 (24.7) | 207 (26.2) |
| For ≥100 days | 781 (69.0) | 570 (72.0) |
| Religion |  |  |
| Hindu | 747 (66.0) | 581 (69.4) |
| Muslim | 244 (21.5) | 231 (27.6) |
| Others | 141 (12.5) | 25 (3.0) |
| Below poverty line (BPL) status |  |  |
| No | 438 (38.7) | 262 (31.3) |
| Yes | 532 (47.0) | 521 (62.3) |
| Not known | 162 (14.3) | 54 (6.4) |
| Parity |  |  |
| Nulliparous | 811 (71.6) | 569 (68.0) |
| Para 1-2 | 276 (24.4) | 242 (28.9) |
| Para ≥3 | 42 (3.7) | 26 (3.1) |
| Missing | 3 (0.3) | 0 (0) |
| Gravidity (including current pregnancy) |  |  |
| Primigravida | 802 (70.8) | 548 (65.5) |
| Gravida 2-3 | 285 (25.2) | 263 (31.4) |
| Gravida >3 | 44 (3.9) | 26 (3.1) |
| Missing | 1 (0.1) | 0 (0) |
| Mode of childbirth |  |  |
| Spontaneous vaginal birth | -- | 580 (69.3) |
| Operative vaginal birth | -- | 174 (20.8) |
| Elective Caesarean Section | -- | 4 (0.5) |
| Emergency Caesarean Section | -- | 79 (9.4) |
| Iron biomarkers | Median (IQR) | Median (IQR) |
| Serum ferritin (in μg/L) | 24.7 (39.4) | 41.9 (59.6)^ǂ^ |
| Soluble transferrin receptor (sTfR in mg/L) | -- | 1.4 (0.9) |
| Transferrin saturation (TSAT in %) | -- | 14.2 (15.3) |
| Serum hepcidin (in ng/ml) | -- | 12.2 (23.7) |
| Ferritin levels^**^ |  |  |
| Low | 351 (31.0) | 306 (36.6) ^ǂ^ |
| Normal | 742 (65.5) | 473 (56.5) ^ǂ^ |
| High | 39 (3.5) | 49 (5.9) ^ǂ^ |
| Missing | 0 (0) | 9 (1.1) ^ǂ^ |
| Soluble transferrin receptor (sTfR) |  |  |
| ≤1.8 mg/L | -- | 556 (66.4) |
| >1.8 mg/L | -- | 250 (29.9) |
| Missing | -- | 31 (3.7) |
| Transferrin saturation (TSAT) |  |  |
| <16% | -- | 459 (54.8) |
| 16-50% | -- | 297 (35.5) |
| >50% | -- | 67 (8.0) |
| Missing | -- | 14 (1.7) |

^$^Abnormal haemoglobin types include HbE trait and homozygous β-thalassemia trait, sickle cell trait and unspecified;  ^*^Vitamin B12 deficiency defined as levels <200ng/L or <148pmol/L; ^**^Antenatal: low <15 μg/L, normal 15-200 μg/L, high >200 μg/L; postnatal: low <30 μg/L, normal 30-200 μg/L, high >200 μg/; ^ǂ^ Measurements adjusted for inflammation; BPL status defined as households (average 5 family members) with per capita consumption expenditure of INR 672.8 on a monthly basis in rural areas and INR 859.6 in urban areas at prices prevailing in 2009-10 (Planning Commission; Government of India, 2012). Note: more primigravida women were excluded than nulliparous women based on inclusion and exclusion criteria, hence the number of primigravida women appear to be lower than nulliparous women.

**Table S2: Descriptive statistics comparing anaemia and iron status in the antenatal cohort**

|  | Anaemia [Frequency (%)] | | | | p-value* |
| --- | --- | --- | --- | --- | --- |
|  | No anaemia | Mild | Moderate | Severe |  |
| Haemoglobin variants |  |  |  |  | <0.001 |
| Normal haemoglobin type | 416 (84.9) | 172 (72.0) | 257 (70.2) | 31 (83.8) |  |
| Abnormal haemoglobin types^$^ | 71 (14.5) | 65 (27.2) | 104 (28.4) | 6 (16.2) |  |
| Missing | 3 (0.6) | 2 (0.8) | 5 (1.4) | 0 (0) |  |
| Ferritin |  |  |  |  | <0.001 |
| <15 μg/L | 85 (17.3) | 77 (32.2) | 168 (45.9) | 21 (56.8) |  |
| 15-200 μg/L | 392 (80.0) | 154 (64.4) | 187 (51.1) | 9 (24.3) |  |
| >200 μg/L | 13 (2.7) | 8 (3.4) | 11 (3.0) | 7 (18.9) |  |

*Chi-square test for difference in proportions; ^$^Abnormal haemoglobin types include HbE trait and homozygous β-thalassemia trait, sickle cell trait and unspecified

**Table S3: Descriptive statistics comparing anaemia and iron status in the postnatal cohort**

|  | Anaemia [Frequency (%)] | | | | p-value* |
| --- | --- | --- | --- | --- | --- |
|  | No anaemia | Mild | Moderate | Severe |  |
| Haemoglobin variants |  |  |  |  | 0.002 |
| Normal haemoglobin type | 276 (84.7) | 167 (76.3) | 181 (69.4) | 25 (80.7) |  |
| Abnormal haemoglobin types^$^ | 47 (14.4) | 51 (23.3) | 76 (29.1) | 6 (19.3) |  |
| Missing | 3 (0.9) | 1 (0.4) | 4 (1.5) | 0 (0) |  |
| Ferritin^ǂ^ |  |  |  |  |  |
| <30 μg/L | 89 (27.3) | 77 (35.2) | 122 (46.7) | 18 (58.1) | <0.001 |
| 30-200 μg/L | 221 (67.8) | 129 (58.9) | 114 (43.7) | 9 (29.0) |  |
| >200 μg/L | 13 (4.0) | 11 (5.0) | 21 (8.1) | 4 (12.9) |  |
| Missing | 3 (0.9) | 2 (0.9) | 4 (1.5) | 0 (0) |  |
| Soluble transferrin receptor (sTfR) |  |  |  |  |  |
| ≤1.8 mg/L | 257 (78.8) | 156 (71.2) | 134 (51.3) | 9 (29.0) | <0.001 |
| >1.8 mg/L | 54 (16.6) | 57 (26.0) | 119 (45.6) | 20 (64.5) |  |
| Missing | 15 (4.6) | 6 (2.7) | 8 (3.1) | 2 (6.5) |  |
| Transferrin saturation (TSAT) |  |  |  |  |  |
| <16% | 185 (56.8) | 120 (54.8) | 135 (51.7) | 19 (61.3) | <0.001 |
| 16-50% | 124 (38.0) | 87 (39.7) | 84 (32.2) | 2 (6.5) |  |
| >50% | 13 (4.0) | 9 (4.1) | 36 (13.8) | 9 (29.0) |  |
| Missing | 4 (1.2) | 3 (1.4) | 6 (2.3) | 1 (3.2) |  |
| Vitamin B12 deficiency |  |  |  |  |  |
| No | 213 (65.3) | 139 (63.5) | 143 (54.8) | 15 (48.4) | 0.021 |
| Yes | 74 (22.7) | 52 (23.7) | 86 (33.0) | 14 (45.2) |  |
| Missing | 39 (12.0) | 28 (12.8) | 32 (12.2) | 2 (6.4) |  |

*Chi-square test for difference in proportions; ^ǂ^Adjusted for inflammation using C-reactive protein; ^$^Abnormal haemoglobin types include HbE trait and homozygous β-thalassemia trait, sickle cell trait and unspecified

**Table-S4: Relationship between anaemia, haemoglobin variants and vitamin B12 deficiency in women with uncomplicated pregnancies**

| Anaemia | Antenatal | | | | | | | Postnatal | | | |
| --- | --- | --- | --- | --- | --- | --- | --- | --- | --- | --- | --- |
|  | cRRR (95% CI) | | p-value | aRRR^*^(95% CI) | | | p-value | cRRR (95% CI) | p-value | aRRR^**^ (95% CI) | p-value |
| **Haemoglobin variants** | | | | | | | | | | | |
| No anaemia | Baseline outcome | | | | | | | Baseline outcome | | | |
| Mild anaemia |  | |  |  | | |  |  |  |  |  |
| Normal Hb type | 1 (Ref) | |  | 1 (Ref) | | |  | 1 (Ref) |  | 1 (Ref) |  |
| Abnormal Hb type | 2.21 (1.51 - 3.24) | | <0.001 | 2.10 (1.42 - 3.09) | | | <0.001 | 1.79 (1.15 - 2.79) | 0.009 | 1.89 (1.17 - 3.08) | 0.010 |
| Moderate anaemia |  | |  |  | | |  |  |  |  |  |
| Normal Hb type | 1 (Ref) | |  | 1 (Ref) | | |  | 1 (Ref) |  | 1 (Ref) |  |
| Abnormal Hb type | 2.37 (1.69 – 3.33) | | <0.001 | 2.30 (1.63 - 3.25) | | | <0.001 | 2.47 (1.64 – 3.71) | <0.001 | 3.03 (1.94 - 4.73) | <0.001 |
| Severe anaemia |  | |  |  | | |  |  |  |  |  |
| Normal Hb type | 1 (Ref) | |  | 1 (Ref) | | |  | 1 (Ref) |  | 1 (Ref) |  |
| Abnormal Hb type | 1.13 (0.46 - 2.82) | | 0.786 | 1.01 (0.40 – 2.54) | | | 0.978 | 1.41 (0.55 - 3.62) | 0.476 | 1.79 (0.61 - 5.23) | 0.290 |
| **Vitamin B12 deficiency**^ǂ^ | | | | | | | | | | | |
| No anaemia |  | | | | | | | Baseline outcome | | | |
| Mild anaemia |  |  | | |  |  | |  |  |  |  |
| Vitamin B12 replete | --- | --- | | | --- | --- | | 1 (Ref) |  | 1 (Ref) |  |
| Vitamin B12 deficient | --- | --- | | | --- | --- | | 1.07 (0.70 - 1.62) | 0.764 | 1.01 (0.64 - 1.59) | 0.959 |
| Moderate anaemia |  |  | | |  |  | |  |  |  |  |
| Vitamin B12 replete | --- | --- | | | --- | --- | | 1 (Ref) |  | 1 (Ref) |  |
| Vitamin B12 deficient | --- | --- | | | --- | --- | | 1.66 (1.14 - 2.44) | 0.009 | 1.75 (1.18 - 2.61) | 0.006 |
| Severe anaemia |  |  | | |  |  | |  |  |  |  |
| Vitamin B12 replete | --- | --- | | | --- | --- | | 1 (Ref) |  | 1 (Ref) |  |
| Vitamin B12 deficient | --- | --- | | | --- | --- | | 2.55 (1.10 - 5.88) | 0.028 | 2.51 (0.95 - 6.59) | 0.063 |

^*^Adjusted for woman’s age and BMI, gestational age at blood measurement, study hospital; ^**^Adjusted for woman’s age, BMI, mode of childbirth, blood loss during childbirth, hours after childbirth at blood measurement, gestational age at childbirth and study hospital. ^ǂ^Both crude and adjusted models for Vitamin B12 deficiency were also adjusted for inflammation using C-reactive protein

Abnormal haemoglobin types include HbE trait and homozygous β-thalassemia trait, sickle cell trait and unspecified

**Table-S5: Relationship between haemoglobin and iron biomarkers stratified by haemoglobin variants**

| **Iron biomarkers** | Haemoglobin variants (antenatal group) | | | |
| --- | --- | --- | --- | --- |
|  | Normal Hb type | | Abnormal Hb types | |
|  | Adj Coef^*^ (95% CI) | p-value | Adj Coef^*^ (95% CI) | p-value |
| Serum ferritin in μg/L | 0.0004  (-0.001 to 0.002) | 0.645 | -0.00006  (-0.001 to 0.001) | 0.927 |
|  | Haemoglobin variants (postnatal group) | | | |
|  | Normal Hb type | | Abnormal Hb types | |
|  | Adj Coef^ǂǂ^ (95% CI) | p-value | Adj Coef^ǂǂ^ (95% CI) | p-value |
| Serum ferritin in μg/L | 0.001 (-0.001 to 0.002) | 0.349 | -0.002 (-0.004 to 0.0002) | 0.078 |
| sTfR in mg/L | -0.03 (-0.06 to -0.01) | 0.012 | -0.44 (-0.63 to -0.25) | <0.001 |
| Serum hepcidin in ng/ml | 0.03 (0.02 to 0.04) | <0.001 | 0.01 (0.003 to 0.02) | 0.013 |

^*^Adjusted for woman’s age and BMI, gestational age at blood measurement, study hospital
^ǂǂ^ Adjusted for woman’s age, BMI, mode of childbirth, blood loss at childbirth, hours after childbirth at blood measurement, gestational age at childbirth, study hospital, and inflammation using C-reactive protein

Abnormal haemoglobin (Hb) types include HbE trait and homozygous β-thalassemia trait, sickle cell trait and unspecified

**Table S6: Association between antenatal anaemia and ferritin levels stratified by haemoglobin variants**

| Antenatal anaemia | Normal Hb type | | Abnormal Hb types | |
| --- | --- | --- | --- | --- |
|  | aRRR^*^ (95% CI) | p-value | aRRR^*^ (95% CI) | p-value |
| No anaemia | Baseline outcome | | | |
| Mild anaemia |  |  |  |  |
| Ferritin <15 μg/L | 2.57 (1.69 – 3.89) | <0.001 | 2.09 (0.80 – 5.43) | 0.132 |
| Ferritin 15-200 μg/L | 1 (Ref) |  | 1 (Ref) |  |
| Ferritin >200 μg/L | 1.81 (0.59 – 5.51) | 0.298 | 1.63 (0.31 – 8.61) | 0.565 |
| Moderate anaemia |  |  |  |  |
| Ferritin <15 μg/L | 5.38 (3.73 – 7.77) | <0.001 | 3.07 (1.30 – 7.28) | 0.011 |
| Ferritin 15-200 μg/L | 1 (Ref) |  | 1 (Ref) |  |
| Ferritin >200 μg/L | 1.31 (0.40 – 4.30) | 0.662 | 1.75 (0.39 – 7.84) | 0.467 |
| Severe anaemia |  |  |  |  |
| Ferritin <15 μg/L | 12.14 (4.97 – 29.6) | <0.001 | 26.1 (2.24 – 304.6) | 0.009 |
| Ferritin 15-200 μg/L | 1 (Ref) |  | 1 (Ref) |  |
| Ferritin >200 μg/L | 24.9 (6.49 – 95.6) | <0.001 | 52.4 (3.33 – 825.3) | 0.005 |

*Adjusted for woman’s age and BMI, gestational age at blood measurement, study hospital; RRR: relative risk ratio; Hb: haemoglobin

Abnormal Hb types include HbE trait and homozygous β-thalassemia trait, sickle cell trait and unspecified

**Table S7: Association between postnatal anaemia and ferritin levels stratified by haemoglobin variants**

| Postnatal anaemia | Normal Hb type | | Abnormal Hb types | |
| --- | --- | --- | --- | --- |
|  | aRRR^ǂǂ^ (95% CI) | p-value | aRRR^ǂǂ^ (95% CI) | p-value |
| No anaemia | Baseline outcome | | | |
| Mild anaemia |  |  |  |  |
| Ferritin <30 μg/L | 1.82 (1.17 – 2.85) | 0.008 | 0.96 (0.35 – 2.64) | 0.932 |
| Ferritin 30-200 μg/L | 1 (Ref) |  | 1 (Ref) |  |
| Ferritin >200 μg/L | 0.41 (0.09 – 1.94) | 0.260 | 1.63 (0.13 – 20.3) | 0.704 |
| Moderate anaemia |  |  |  |  |
| Ferritin <30 μg/L | 3.62 (2.34 – 5.60) | <0.001 | 0.99 (0.39 – 2.52) | 0.984 |
| Ferritin 30-200 μg/L | 1 (Ref) |  | 1 (Ref) |  |
| Ferritin >200 μg/L | 1.43 (0.46 – 4.45) | 0.537 | 6.31 (0.70 – 56.7) | 0.101 |
| Severe anaemia |  |  |  |  |
| Ferritin <30 μg/L | 5.23 (1.61 – 17.04) | 0.006 | Very small – not calculated | -- |
| Ferritin 30-200 μg/L | 1 (Ref) |  | 1 (Ref) |  |
| Ferritin >200 μg/L | 3.20 (0.27 – 37.1) | 0.353 | 58.6 (1.94 – 1771.3) | 0.019 |

^ǂǂ^ Adjusted for woman’s age, BMI, mode of childbirth, blood loss at childbirth, hours after childbirth at blood measurement, gestational age at childbirth, study hospital, and inflammation using C-reactive protein; RRR: relative risk ratio; Hb: haemoglobin

Abnormal Hb types include HbE trait and homozygous β-thalassemia trait, sickle cell trait and unspecified
